# A multiple comparative study of putative endosymbionts in three coexisting apple snail species

**DOI:** 10.7717/peerj.8125

**Published:** 2019-12-06

**Authors:** Federico A. Dellagnola, Cristian Rodriguez, Alfredo Castro-Vazquez, Israel A. Vega

**Affiliations:** IHEM, CONICET, Universidad Nacional de Cuyo, Mendoza, Argentina; Universidad Nacional de Cuyo, Facultad de Ciencias Médicas Instituto de Fisiología, Mendoza, Argentina; Universidad Nacional de Cuyo, Facultad de Ciencias Exactas y Naturales, Departamento de Biología, Mendoza, Argentina

**Keywords:** Ampullariidae, *Pomacea*, *Asolene*, Intracellular bacteria, Endosymbiosis, Coevolution, Digestive gland, Morphometry, 16S rRNA, Fluorescence in situ hybridisation, Lysozyme sensitivity

## Abstract

We here compare morphological and molecular characters of some putative endosymbiotic elements of the digestive gland of three ampullariid species (*Pomacea canaliculata*, *Pomacea scalaris* and *Asolene platae*) which coexist in Lake Regatas (Palermo, Buenos Aires). The putative endosymbionts were reported in these species and were identified as C and K corpuscles. The three species show tubuloacinar glands, each adenomere was constituted mainly by two distinct cell types (columnar and pyramidal). C and K corpuscles together occupied from one-fourth to one-fifth of the tissue area in the three host species, where C corpuscles were round and greenish-brown, were delimited by a distinct wall, stained positively with Alcian Blue and were associated with columnar cells. K corpuscles were oval, dark-brown multilamellar bodies and were associated with pyramidal cells. Under TEM, C corpuscles occurred within vacuoles of columnar cells and contained many electron-dense clumps and irregular membrane stacks and vesicles spread in an electron-lucent matrix. Sometimes a membrane appeared detached from the inner surface of the wall, suggesting the existence of a plasma membrane. In turn, K corpuscles were contained within vacuoles of pyramidal cells and were made of concentric lamellae, which were in turn made of an electron-dense fibrogranular material. No membranes were seen in them. Interspecifically, C corpuscles vary significantly in width and inner contents. K corpuscles were also variable in length and width. However, both C and K corpuscles in the three studied species hybridised with generalised cyanobacterial/chloroplast probes for 16S rRNA. Also, both corpuscle types (isolated from gland homogenates) were sensitive to lysozyme digestion, which indicates that bacterial peptidoglycans are an integral part of their covers. The reported data confirm and extend previous studies on *P. canaliculata* in which the endosymbiotic nature of C and K corpuscles were first proposed. We further propose that the endosymbiotic corpuscles are related to the Cyanobacteria/chloroplasts clade. Based on the known distribution of these corpuscles in the major clades of Ampullariidae, we hypothesise they may be universally distributed in this family, and that may constitute an interesting model for studying the co-evolution of endosymbionts and their gastropod hosts.

## Introduction

Apple snails (Ampullariidae) are a family of architaenioglossan caenogastropods that originated in the Gondwana supercontinent (Berthold, 1991), and most extant species are known for Neotropical, African and Asian regions (Hayes et al., 2015). Light microscopy studies have shown that pigmented intracellular corpuscles occur in the digestive gland of ampullariids in the Neotropical genera *Asolene*, *Felipponea*, *Marisa* and *Pomacea* (Castro-Vazquez et al., 2002; Dellagnola, Castro-Vazquez & Vega, 2016), in African and Asian species of *Pila* (Meenakshi, 1955; Devi, Rao & Shyamasundari, 1981; K Ademolu & A Castro-Vazquez, pers. comm., 2018), and in the African genus *Lanistes* (K Ademolu & A Castro-Vazquez, pers. comm., 2018), i.e., in the main clades of the family Ampullariidae (Hayes, Cowie & Thiengo, 2009).

In general, the studies in the past century, based on rather simple light microscopy observations, had considered the corpuscles as excretory products of intracellular digestion. However, Castro-Vazquez et al. (2002) offered a different perspective on these bodies and hypothesised the corpuscles could be endosymbionts living within the cells of the digestive gland. The hypothesis arose from their transmission electron microscopy observations and their finding of a bacterium-like DNA/protein ratio in C corpuscles isolated from the digestive gland of *Pomacea canaliculata*. Several studies by using different methodological approaches have followed that proposition (Campoy-Diaz et al., 2018; Godoy, Castro-Vazquez & Vega, 2013; Koch et al., 2006; Koch, Vega & Castro-Vazquez, 2017; Vega et al., 2012a; Vega et al., 2012b; Vega et al., 2005).

Two corpuscle types (termed C and K corpuscles) have been distinguished by Castro-Vazquez et al. (2002). C corpuscles are greenish-brown, round bodies, and have been proposed to be the vegetative forms of the endosymbiont, while K corpuscles are dark brown, oval bodies, and have been proposed to be the cystic form of the same endosymbiont. C corpuscles seem to be transmitted maternally in *P. canaliculata* (Koch, Vega & Castro-Vazquez, 2017), and both C and K corpuscles have been shown to play roles in protein digestion (Godoy, Castro-Vazquez & Vega, 2013) and metal accumulation and depuration (Campoy-Diaz et al., 2018; Vega et al., 2012a) in this species. Most significantly, the 16S rRNA gene can be amplified from template DNA of both corpuscle types (Vega et al., 2005).

At least in the Neotropical genera *Pomacea* and *Asolene*, both C and K corpuscles are released from the digestive gland cells into the gut lumen and are later expelled in the faeces (Castro-Vazquez et al., 2002). C (but not K) corpuscles may persist for years in sediments of aquaria that had contained *P. canaliculata*, indicating they may undergo part of their life cycle in the environment (Koch et al., 2006).

Three ampullariid species (*P. canaliculata*, *Pomacea scalaris* and *Asolene platae*) coexist in Lake Regatas, a lentic water body in Palermo (Buenos Aires). In this paper, we were particularly interested in exploring whether horizontal (= lateral) or maternal (= vertical) transmission of the endosymbiont was predominant in the hosts’ populations. Therefore, if the endosymbiont underwent part of its life cycle in the environment, and if lateral (= horizontal) transmission predominated between the three host populations, endosymbionts of similar characteristics would be present in the digestive glands of the three ampullariid species. Alternatively, if the symbiotic association had occurred in an ancestor of these species, and if some form of maternal (= vertical) transmission were predominant, the endosymbionts would have diverged during coevolution with the ampullariid hosts.

To explore these possibilities, we have compared morphological and molecular characters of the endosymbionts (as well as their cellular associations) in the three ampullariid species that coexist in Lake Regatas, using (1) light microscopy (LM) of trichrome-stained sections and morphometry of the putative endosymbionts and related glandular structures, (2) transmission electron microscopy (TEM) of C and K corpuscles in the three host species, (3) fluorescence *in situ* hybridisation (FISH) with a generalised cyanobacterial 16S rRNA probe, and (4) a lysozyme test for bacterial peptidoglycans in the corpuscles covers.

## Materials and Methods

### Collection site

Individuals of *P. canaliculata*, *P. scalaris*, and *A. platae* were collected in Lake Regatas (34°33′19.65 ″S, 58° 26′4.33 ″W) during January 2011 and 2017 (Southern summer). This lentic water body occasionally communicates with the Plata river and its riverine flora is dominated by grasses, shrubs, and trees (Lahitte et al., 1998). No submerse or emerse macrophytes were observed in the lake at the times of sampling. Besides the three ampullariid species that are the objects of this study, the molluscan fauna included gastropods in the genera *Chilina* (Chilinidae), *Biomphalaria* (Planorbidae) and *Heleobia* (Hydrobiidae), as well as the invasive bivalve *Corbicula fluminea* (Cyrenidae).

### Sacrifice, sampling and fixation for light microscopy (LM), fluorescence in situ hybridisation (FISH) and transmission electron microscopy (TEM)

Faecal material eliminated by each snail during the first 30 min after removal from the lake was fixed in 4% paraformaldehyde and carried to the laboratory in Mendoza for LM observations. Three to five individuals of each species were put into an ice bath for at least 15 min (both for relaxation and minimising pain), after which the shell was cracked, and the digestive gland was dissected out and divided into small pieces with a razor blade (about three mm^3^ blocks for LM and FISH, and one mm^3^ blocks for TEM). The LM samples were fixed in 4% paraformaldehyde, while those for TEM were fixed in Karnovsky solution (2.5% glutaraldehyde, 4% paraformaldehyde). All fixatives were made in a buffered solution (*Pc*ABS: 43mM NaCl, 1.8 mM KCl, 10 mM HEPES and 30 mM EDTA; pH 7.6; Cueto et al., 2015) that was designed to match the normal plasma osmolality and pH of *P. canaliculata* (Cueto et al., 2011).

### Preparation for LM

Fixed samples of the digestive gland were dehydrated in a graded ethanol series followed by xylene and were then embedded in a resin-paraffin mixture (Histoplast^®^). Sections (5 µm thick, at least 25 µm apart) were stained with a trichrome stain (Nuclear Fast Red, Alcian Blue 8GX, eosin; Dellagnola, Vega & Castro-Vazquez, 2017), in which nuclei were stained bright red, glycosaminoglycans were stained deep blue and cytoplasm was stained from light blue to purple. Micrographs were taken with a Nikon Eclipse 80i (using either bright field or Nomarski differential-interference contrast (DIC) microscopy) provided with a Nikon DS-Fi1-U3 digital camera.

### Morphometry performed on gland sections and isolated C and K corpuscles

The tissue area occupied by columnar cells, pyramidal cells, C and K corpuscles, as well as the mean total area occupied by the adenomeres was determined on micrographs of gland sections obtained from three individuals of each species (10 replicates from each individual). Measurements were made using Image-Pro Plus, version 6.0.0.260. Finally, the mean values of each parameter were computed for each snail and used for further statistical analysis.

C and K corpuscles isolated from digestive gland tissue (according to Vega et al., 2005) were measured using ImageJ 1.47V (Wayne Rasband National Institutes of Health, USA) and evaluated. A single diameter was measured in the spheroidal C corpuscles, while both the length and width of the oval K corpuscles were measured. The corpuscles were isolated from three individuals per species, and the number of corpuscles measured per species are indicated in Table 1.

**Table 1:** Measurements of C and K corpuscles isolated from digestive glands of the three species. Results are expressed as mean ± SEM. N for each group is indicated in italics. Different letters in the same row indicate statistically significant differences between species.

	***Pomacea canaliculata***	***Pomacea scalaris***	***Asolene platae***
**C corpuscles**			
**Diameter (µm)**	13.8 ± 0.1; *197*^a^	11.6 ± 0.1; *241*^b^	16.5 ± 0.2; *161*^c^
**K corpuscles**			
**Length (µm)**	31.8 ± 0.4; *161*^a^	24.3 ± 0.2; *245*^b^	32.4 ± 0.2; *444*^a^
**Width (µm)**	12.8 ± 0.2; *161*^a^	16.2 ± 0.1; *245*^b^	19.3 ± 0.2; *444*^c^
**Length/width ratio**	2.5 ± 0.05; *161*^a^	1.5 ± 0.01; *245*^b^	1.7 ± 0.02; *444*^c^

### Preparation for TEM

After fixation, the samples were washed three times with *Pc* ABS and transferred to 1% osmium tetroxide overnight. Afterwards, they were rinsed in distilled water and treated with an aqueous solution of 2% uranyl acetate for 40 min, dehydrated in a graded ethanol series followed by acetone and finally embedded in Spurr’s resin. Semi-thin sections stained with toluidine blue were used for topographical orientation. Ultra-thin, silver-grey sections mounted on copper grids were stained with uranyl acetate and lead citrate and examined with a Zeiss EM 900 transmission electron microscope.

### Preparation for FISH using a cyanobacterial/chloroplast 16S rRNA probe

We used a digoxigenin-labelled probe CYA361 [5′-CCCATTGCGGAAAATTCC-3] that recognises a conserved 16S rRNA sequence of Cyanobacteria (Schönhuber et al., 1999) and chloroplasts (Dellagnola, 2010). Tissues were prepared as for LM, and the sections were rehydrated after resin-paraffin removal and then subjected to the following stepwise procedure: (1) incubation in a 2X SSPE hybridisation buffer (1X SSPE = 0.15 M NaCl, 0.01 M EDTA, 0.01 M sodium phosphate, pH 7.2) at 70 °C for 20 min; (2) incubation in 0.1 M triethanolamine solution containing 0.25% acetic anhydride for 10 min; (3) exposure to a 2X SSPE hybridisation buffer containing herring sperm DNA (0.5 mg/ml), yeast tRNA (0.25 mg/ml) and 5X Denhardt’s solution for 60 min at 42 °C; (4) incubation with 100 pmol of the digoxigenin-labelled probe per tissue section, at 37  °C overnight in a humid chamber; (5) sequential washing in decreasing concentrations of the hybridisation buffer (2X, 1X, and 0.5X SSPE, 60 min each) at room temperature; (6) incubation in a buffer containing 100 mM Tris (pH 7.5), 150 mM NaCl and 1% goat serum for 5 min. Afterwards, for detection of the digoxigenin-labelled probe, sections were incubated for 5 h in darkness with a 1:4 dilution of a fluorescein-attached antibody against digoxigenin (Roche, catalogue number 11207741910) and were then washed in a buffer containing 100 mM Tris (pH 7.5) and 100 mM NaCl (three times, 10 min each). Finally, the sections were mounted in glycerol–PBS buffer (90:10; v/v) containing 5 mg/ml propyl-gallate (P3130, Sigma) (Longin et al., 1993). Negative controls (i.e., sections exposed to the CYA361 probe, but with no digoxigenin label) were also run. Observations were made with differential interference contrast (DIC) and fluorescence microscopy (excitatory wavelength range = 465–495 nm; emission wavelength range = 515–555 nm).

### Lysozyme sensitivity

C and K corpuscles from *P. canaliculata*, *P. scalaris* and *A. platae* were used to determine the corpuscles sensitivity to lysozyme (EC 3.2.1.17), an enzyme which catalyses the hydrolysis of 1,4-β-linkages between N-acetyl muramic acid and N-acetyl-D-glucosamine residues in bacterial peptidoglycans (https://www.qmul.ac.uk/sbcs/iubmb/enzyme/EC3/2/1/17.html).

Both C and K corpuscle fractions were obtained from the digestive gland according to Vega et al. (2005) from four individuals of each species (collection date: February 20, 2017; processing date: February 24, 2017). Each corpuscle fraction was washed three times in mannitol-phosphate buffer (0.14 M, pH 6.0), centrifuged (6,000 rpm, 5 min) and suspended in 250 µl of mannitol-phosphate buffer per aliquot (*N* = 5). Fifty microlitres of either MiliQ^®^ water (control) or lysozyme solution (Sigma-L3790, 50 µg/µl) were added to each aliquot and the mixtures were incubated for 1 h at room temperature. Drops of each incubate were observed using DIC microscopy. Then, each aliquot was analysed by flow cytometry (FACS Aria III flow cytometer, BD Bioscience, California, USA).

### Flow cytometry, cell sorting and quantification of lysozyme effect

Both fractions containing C or K corpuscles were identified by flow cytometry and cell sorting. Dot plots of forward light scatter (FSC) and side light scatter (SSC) were used, indicating differences in corpuscle size and complexity-granularity, respectively; 30,000 events per corpuscle incubate were recorded (Fig. S1A). Then, doublets were excluded using forward-scattered light area (FSC-A) and width (FSC-W). Finally, the remaining dots were plotted according to autofluorescence in channels 1 (660 nm) and 2 (695 nm), which gave optimal separations of C and K corpuscles (Fig. S1B), as controlled by light microscopy of the sorted regions (Figs. S1C–S1F). Data analyses were made using FlowJo 7.6.2 software. Results were expressed as the percentage of C, K or C+K corpuscles that appeared lysed after control and lysozyme treatment.

### Statistical analyses

Data analysis was performed using GraphPad Prism, version 6.01. Multigroup comparisons were made using Kruskal–Wallis one-way analysis of variance, followed by the Dunn’s test. Significance level was fixed at *P* < 0.05. Confidence intervals (95%) were used to assess the significance of differences in flow-cytometry experiments.

### Ethical aspects

Procedures for sacrifice were approved by the Institutional Committee for the Care and Use of Laboratory Animals (Facultad de Ciencias Médicas, Universidad Nacional de Cuyo), Approval Protocol N^∘^ 55/2015.

## Results

### Light microscopy

**Figure 1:**
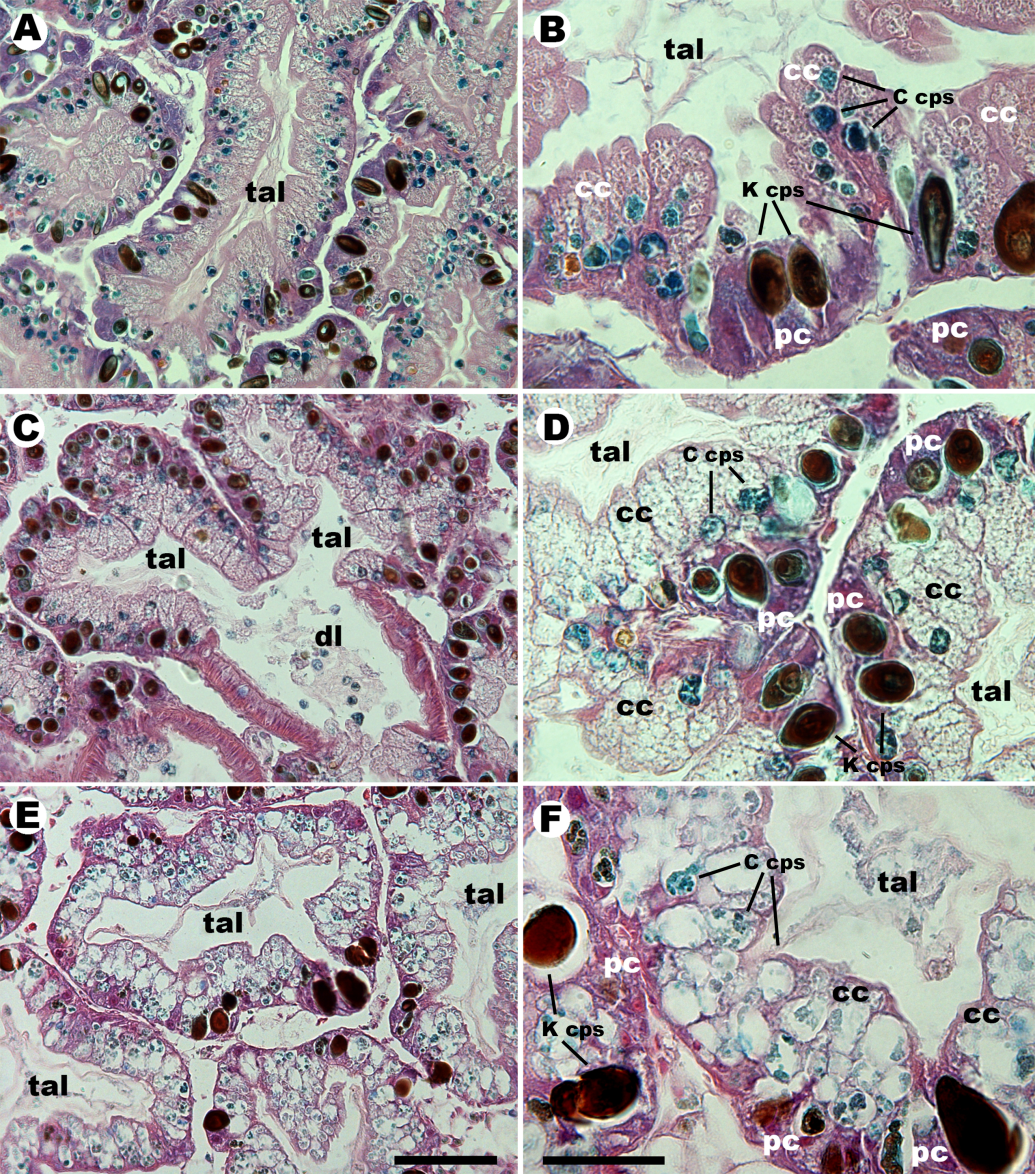
Histology of the digestive gland of ampullariids. Tubuloacini of *Pomacea canaliculata* (A, B), *Pomacea scalari*s (C, D) and *Asolene platae* (E, F), at two different magnifications (trichrome stain). Numerous C corpuscles, containing alcianophilic clumps, appear associated with columnar cells in the three studied species, mostly in their basal region. The dark K corpuscles appear associated with the purple cytoplasm of pyramidal cells. C corpuscles appear definitely larger and clearer in *A. platae* than those in the other two species and, in this section, they extend to the apical region of columnar cells. Ductal branches, lined by a ciliated columnar epithelium with some goblet cells, are more extensive in *P. scalaris* than in the other two species (free C corpuscles are seen within the ductal lumen in panel (C). Abbreviations: cc, columnar cells; C cps, C corpuscles; dl, ductal lumen; K cps, K corpuscles; pc, pyramidal cells; tal, tubuloacinar lumen. Scale bars represent 50 µm for (A, C and E), and 20 µm for (B, D and F).

In trichrome stained sections, the three species showed tubuloacinar digestive glands (Fig. 1). The tubuloacini (i.e., the adenomeres) were composed mainly of columnar and pyramidal cells as those previously described for *P. canaliculata* (Koch et al., 2006). Secretory ducts lined by a ciliated columnar epithelium with some goblet cells were seen in all species but were more extended and ramified in *P. scalaris*. Non-glandular cells and tissues, such as storing cells (Vega et al., 2007), other connective tissue, haemocoelic spaces and vessels were also seen.

Columnar cells were elongated, vacuolated, and contained C corpuscles, which showed alcianophilic inner clumps and covers. Pyramidal cells were triangular, with a purple cytoplasm and a conspicuous red nucleus, and were usually associated with the darkly pigmented K corpuscles.

Morphometric analysis of glandular sections from the three species is summarised in Table 2. The percent area of sections occupied by tubuloacini was similar in *P. canaliculata* and *A. platae*, but it was significantly lower in *P. scalaris* due to the larger development of the ductal system. Columnar and pyramidal cells occupied ∼55% and ∼40% of the tubuloacinar area, with no significant differences between the three species. The occupancy by C corpuscles ranged from 11.4% to 13.3% (being *A. platae  > P. scalaris*). The occupancy by K corpuscles ranged from 11% to 11.4% and there were no significant differences between species. Also, C and K corpuscles were found in the faeces of the three species.

**Table 2:** Percent area of digestive gland sections occupied by tubuloacini, glandular cell types, and C and K corpuscles. Results are expressed as mean percent area ± SEM. Different letters in the same row indicate statistically significant differences between species.

	***Pomacea canaliculata***	***Pomacea scalaris***	***Asolene platae***
Tubuloacini (%)	90.7 ± 0.9^a^	85.1 ± 1.2^b^	92.8 ± 0.9^a^
Columnar cells (%)	53.7 ± 1.7^a^	55.3 ± 1.6^a^	56.3 ± 1.5^a^
Pyramidal cells (%)	41.9 ± 1.5^a^	39 ± 1.4^a^	39.5 ± 1.4^a^
C corpuscles (%)	12.3 ± 0.6^ab^	11.4 ± 0.4^a^	13.3 ± 0.5^b^
K corpuscles (%)	11 ± 0.5^a^	11.4 ± 0.8^a^	11.2 ± 0.5^a^

C corpuscles isolated from digestive gland homogenates were generally round and delimited by a distinct wall in the three studied species. As in previous studies on *P. canaliculata* (Koch et al., 2006; Vega et al., 2005), the content of C corpuscles varied to some extent. In general, those of *P. canaliculata* contained small, irregular, greenish-yellow clumps, while those of *P. scalaris* were brownish and darker; again, those of *A. platae* differed, showing large yellowish-green clumps in an abundant matrix. Mean width of isolated C corpuscles varied significantly between species (Table 1, *A. platae* > *P. canaliculata* > *P. scalaris*).

Otherwise, isolated K corpuscles were generally oval, multilamellar dark bodies, which varied significantly in length (*A. platae* = *P. canaliculata* > *P. scalaris*)*.* There were also significant differences in both width (*A. platae* > *P. scalaris* >*P. canaliculata*) and in the length/width ratio (*P. canaliculata* > *A. platae* > *P. scalaris*). These data are also summarised in Table 1.

### Transmission electron microscopy

**Figure 2:**
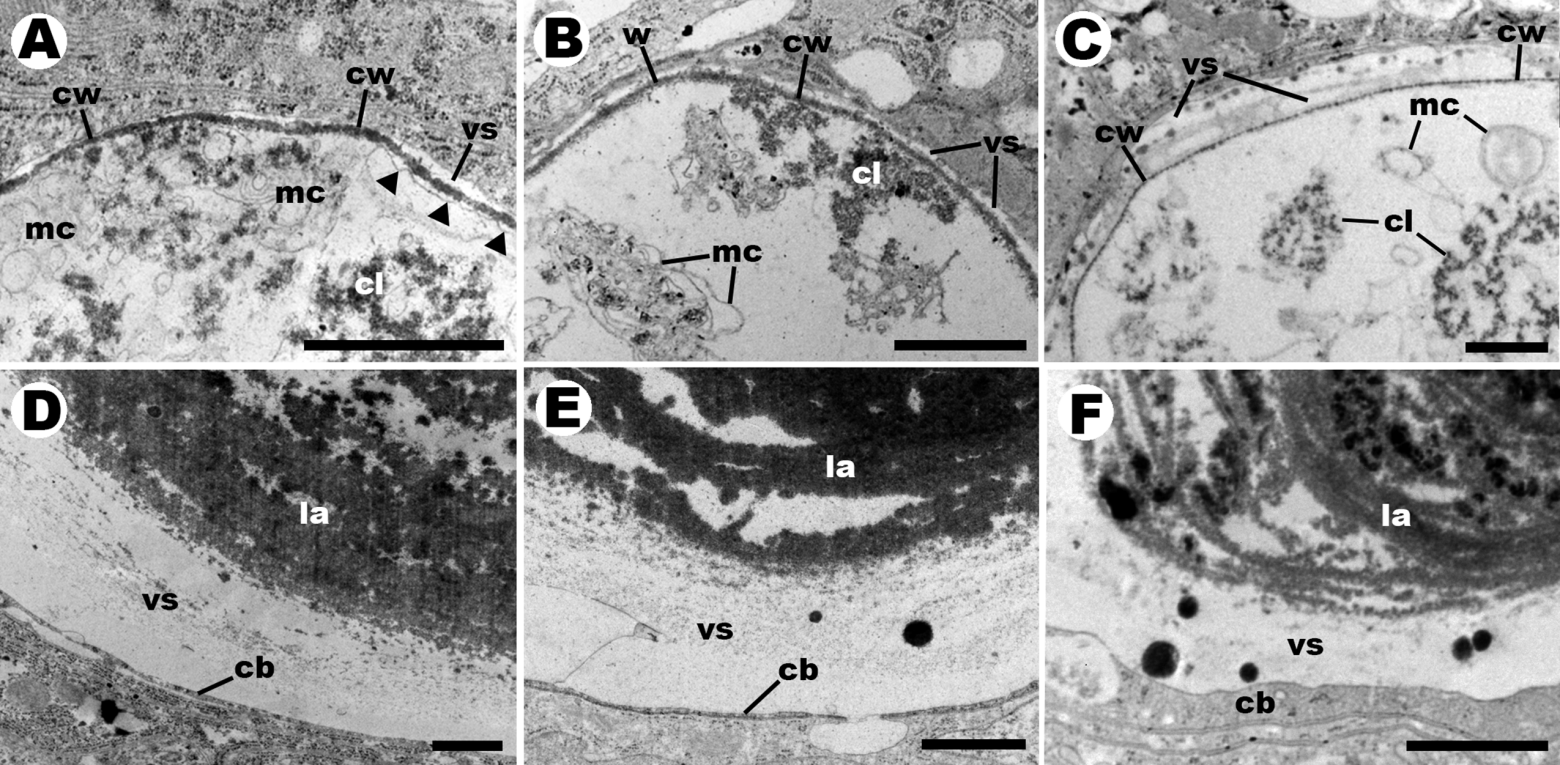
Details of endosymbiotic corpuscles (transmission electron microscopy). C corpuscles in *Pomacea canaliculata* (A), *P. scalaris* (B), and *Asolene platae* (C), respectively. They show an electron-lucent matrix containing many electron-dense clumps and some irregular and often concentric membrane vesicles. C corpuscles are delimited by an electron-dense wall, which seldom shows small pores (only shown in C, for *A. platae*. K corpuscles in *P. canaliculata* (D), *P. scalaris* (E), and *Asolene platae* (F), respectively. They show multiple concentric layers of a fibro-granular material. The vacuoles containing K corpuscles frequently show large globules of electron-dense material. Abbreviations: cb, host cytoplasmic band; cl, electron-dense clump; la, lamella; mc, membrane complex; vs, vesicular space; cw, corpuscular wall. Black triangles indicate zones of detachment of the wall, suggesting a plasma membrane. Scale bars represent 1 µm.

C corpuscles of the three species (Figs. 2A–2C) were contained within vacuoles of columnar cells and were encased in an electron-dense wall, which occasionally showed small pores. The contents showed electron-dense clumps and irregular arrangements of inner membranes in an electron-lucent matrix, but no nuclei or thylakoid structures could be recognised. Sometimes a membrane appeared partly detached from the wall, suggesting the existence of a plasma membrane. C corpuscles in *A. platae* (Fig. 2C) showed clumps larger than those in the two *Pomacea* species and were dispersed in a more abundant matrix. Also, pores in the wall occurred more frequently in *A*. *platae*.

All K corpuscles (Figs. 2D–2F) showed multiple, concentric lamellae made of a fibrogranular material. No nuclei or membranous structures could be recognised in these corpuscles. Also, the vacuoles containing K corpuscles frequently showed electron-dense globules of varying size.

### FISH for cyanobacterial 16S rRNA

**Figure 3:**
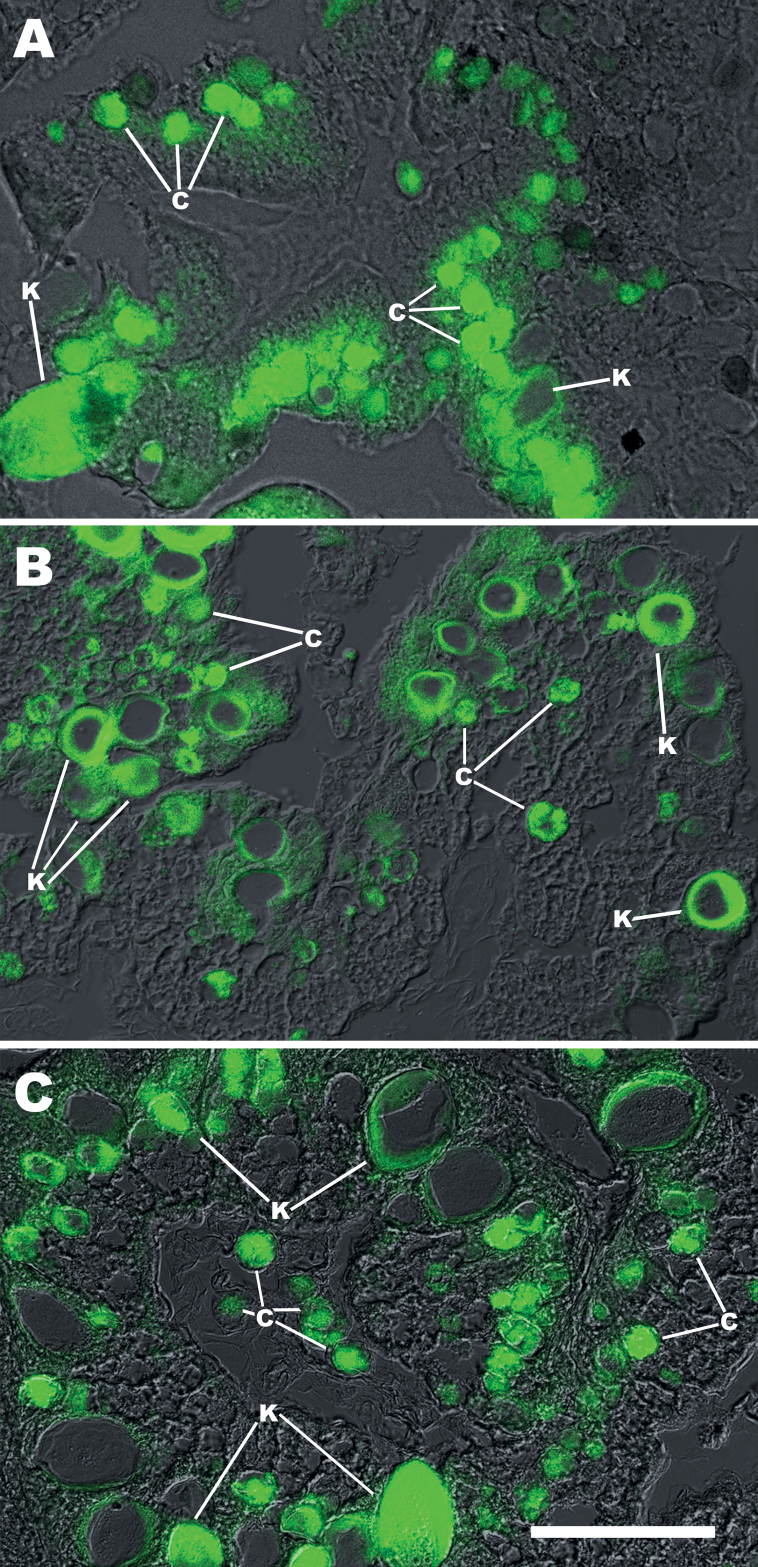
FISH with a generalised cyanobacterial 16S rRNA probe (CYA361) on C and K corpuscles. Merged DIC and fluorescence micrographs on digestive gland of (A) *Pomacea canaliculata*; (B) *Pomacea scalaris*; (C) *Asolene platae*. The central part of K corpuscles is frequently detached during the hybridisation procedure. Abbreviations: c; C corpuscles; c*, free C corpuscles in the tubuloacinar lumen; k, K corpuscles; tal, tubuloacinar lumen. Scale bar represents 50 µm for (A–C).

Sections of the digestive gland exposed to the digoxigenin-labelled CYA361 probe (Fig. 3) showed fluorescence in most C and K symbiotic corpuscles. However, the cores of some K corpuscles were detached and lost during the hybridisation procedure. Sections exposed to probes that were not labelled with digoxigenin (negative controls) showed only some background fluorescence (Fig. S2).

### Lysozyme sensitivity (DIC & flow cytometry)

**Figure 4:**
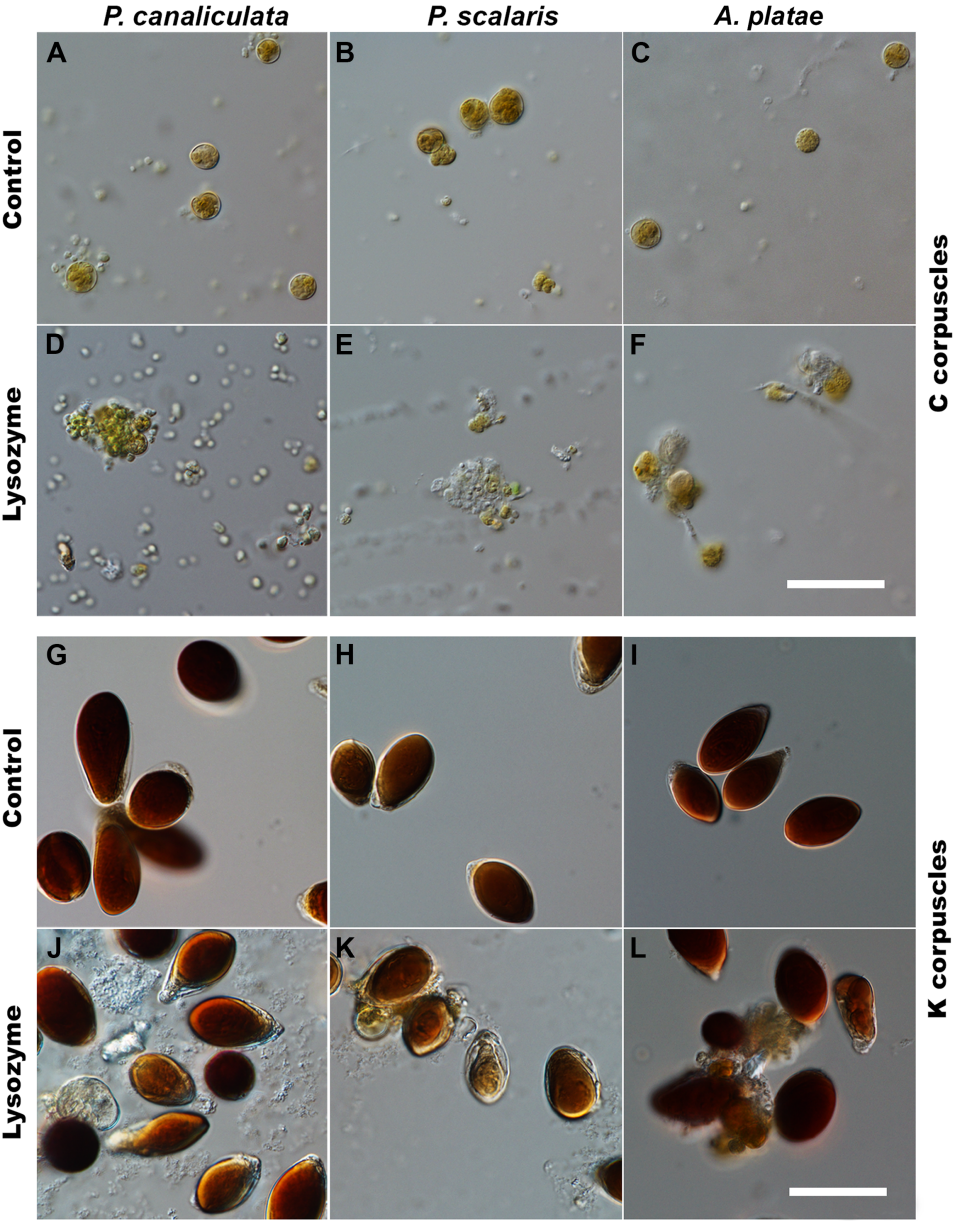
Effect of lysozyme digestion on isolated C and K corpuscles in *Pomacea canaliculata*, *Pomacea scalaris* and *Asolene platae* (DIC microscopy). (A–C) Suspension of C corpuscles that were not exposed to the enzyme. (D–F) Lysozyme-treated suspensions of C corpuscles, showing debris, partial lysis and aggregation of corpuscles. (G–I) Suspension of K corpuscles that were not exposed to the enzyme. (J–L) Lysozyme-treated suspensions of K corpuscles. Scale bars represent 30 µm.

Enzyme treatment resulted in partial degradation of both C and K corpuscles (Fig. 4). DIC microscopy of treated suspensions showed that the remaining corpuscles and debris became aggregated in masses (C corpuscles) or showed spreading of material into the medium (K corpuscles).

**Figure 5:**
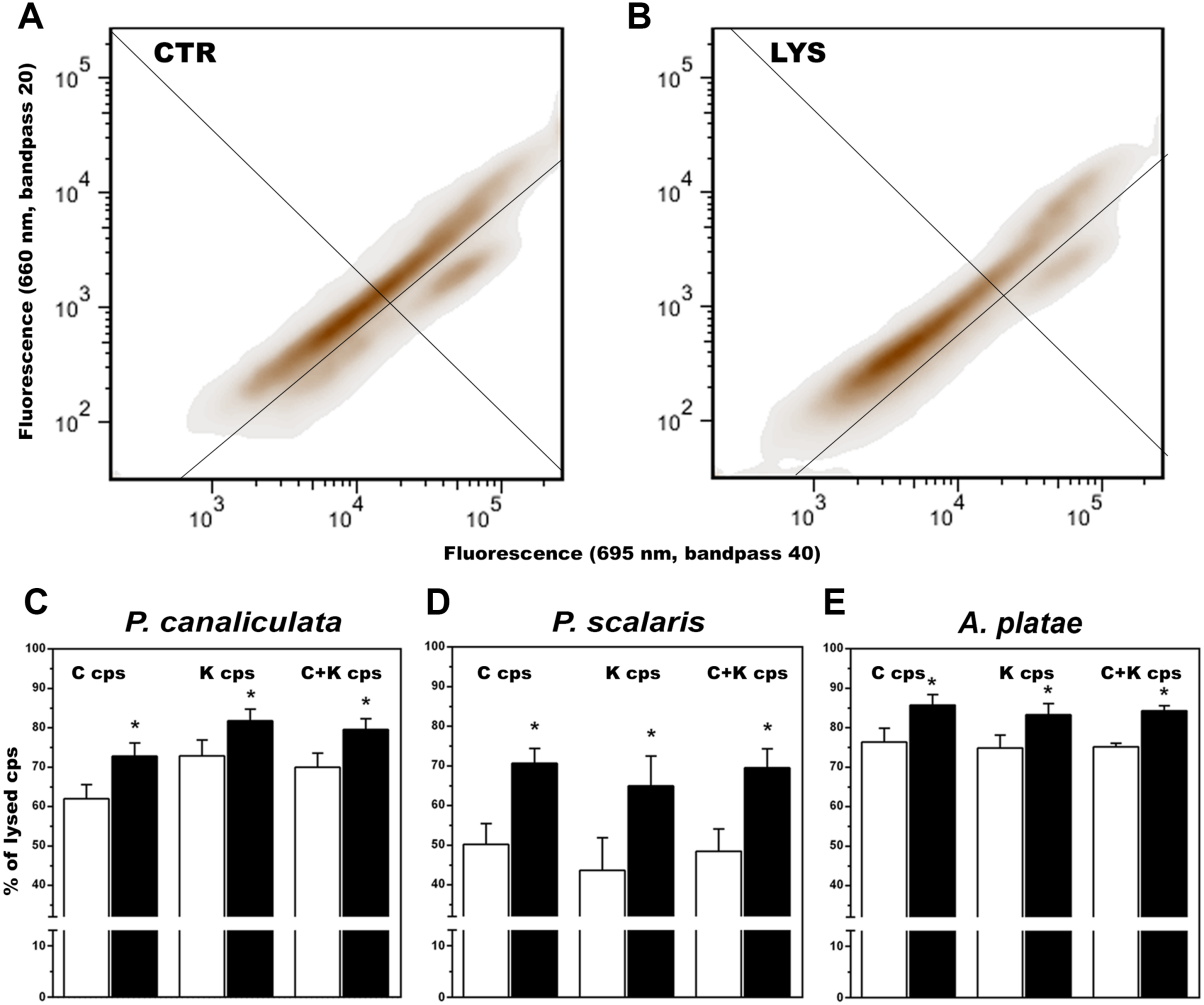
Quantification by flow cytometry of the effect of lysozyme digestion on isolated C and K corpuscles of *Pomacea canaliculata*, *P. scalaris* and *Asolene platae.* (A–B) Dot plots of a representative assay of corpuscles isolated from *P. canaliculata*, using two fluorescence channels (660 nm and 695 nm); the same corpuscular suspension was divided into control (A) and lysozyme-treated (B) aliquots. In the upper frame are undamaged K corpuscles, whereas in the right frame are undamaged C corpuscles. (C–E) Percent of lysed C and K corpuscles after lysozyme treatment, as compared with their respective controls, in the three studied species (30,000 events per aliquot; four snails were studied per species, and C and K aliquots were studied per snail). White and black bars represent control and lysozyme-treated corpuscles, respectively (mean ±  SEM). Asterisks indicate a statistically significant difference from the respective control (95% confidence limits).

The effect of lysozyme treatment of isolated C and K corpuscles was quantified by flow cytometry (Figs. 5A–5B). In the three studied species, the percent of lysed C and K corpuscles increased significantly after lysozyme treatment (Figs. 5C–5E).

## Discussion

### The digestive gland and its putative endosymbionts

Meenakshi (1955) was the first to mention pigmented corpuscles in the digestive gland of an ampullariid (*Pila virens*) and those corpuscles were probably of the type we are here referring to as K corpuscles. Later, Andrews (1965) reported both corpuscle types in the digestive gland and faeces of *P. canaliculata,* and she identified them as ‘greenish spherules’ and ‘dark concretions’, corresponding to our C and K corpuscles, respectively. Both authors interpreted the corpuscles as digestive or excretory bodies.

Indeed, at first glance, both types could be interpreted as ‘residual bodies’ (Wartosch, Bright & Luzio, 2015) resulting from the intracellular digestion of algal or plant chloroplasts. However, C corpuscles also appear in the glands of juveniles of *P. canaliculata* that have not eaten any algal or plant material (Koch, Vega & Castro-Vazquez, 2017), which indicates they are not remnants of chloroplast digestion. Also, C corpuscles are about 12–17 µm large while chloroplasts are 5–10 µm large (Cooper & Hausman, 2000; Milo & Phillips, 2015); furthermore, C corpuscles are larger than any structure of the endocytic pathway that has been reported in gastropods (Lobo-da Cunha, 2000; Marigómez et al., 2002; Ojeda, Arrighetti & Giménez, 2015; Taïeb, 2001) and they are also larger than most bacteria, with the exception of some genera of Pleurocapsales (Cyanobacteria; (Waterbury, 1989)), and one extreme case within the Gammaproteobacteria (Proteobacteria; (Schulz, 2006)).

C corpuscles in *P. canaliculata* show (1) no nucleus (Castro-Vazquez et al., 2002); (2) chlorophyll-like pigments (phoeophorbides *a* and *b*), but no phycobilins (Vega et al., 2012b) or thylakoids, although they show irregularly arranged stacks of inner membranes (Koch et al., 2006); (3) an electron-dense wall (Koch et al., 2006; Vega et al., 2005); (4) a plasma membrane with the typical lipid bilayer, which is sometimes seen when it is detached from the electron-dense wall (Koch et al., 2006); and (5) inner electron-dense clumps but no organelles (Koch et al., 2006; Vega et al., 2005). Furthermore, they are eliminated in the faeces and are found for years in sediments of aquaria that had contained *P. canaliculata* (Koch et al., 2006), which suggests that they may reproduce there. Together, these findings agree with the proposal (Castro-Vazquez et al., 2002) that C corpuscles are prokaryotic endosymbionts in digestive gland cells, which are released to the environment, where they may undergo part of their life cycle.

On their part, K corpuscles are multilayered structures of an electron-dense material that encloses a core that chips away when cutting with the diamond knife. Apparently, K corpuscles are difficult to penetrate by the fixative and embedding media, and this may explain why membranes are not observed, because they would be lost in the course of these procedures. An alternative (and parsimonious) interpretation would be that membranes never existed in K corpuscles, and consequently, that these bodies are not a cystic form of C corpuscles, as proposed by Castro-Vazquez et al. (2002), but some product of digestion. However, the hypothesis of a dietary origin of K corpuscles is difficult to reconcile with their hybridisation with a generalised cyanobacterial/chloroplast probe for 16S rRNA (Fig. 3) and with their sensitivity to lysozyme digestion (Figs. 4 and 5). Therefore, we must conclude that the nature of K corpuscles is still an unresolved matter.

Pigmented corpuscles similar to C and K corpuscles of Ampullariidae have also been shown in another, phylogenetically distant gastropod (*Lobatus gigas*, Littorinimorpha, Strombidae; (Dellagnola, Vega & Castro-Vazquez, 2017). The exact correspondence of the corpuscles found in *L. gigas* with those of Ampullariidae would have to be explored. However, their occurrence in a littorinimorph suggests that similar endosymbiotic associations may have occurred more than once in the evolution of gastropods.

### Comparative characters of C and K corpuscles in ampullariids coexisting in Lake Regatas

As in the previous study in *P. canaliculata* (Koch et al., 2006), C corpuscles in the other two species were associated with columnar cells of the digestive gland (Fig. 1), but they differed significantly in width (Table 1; *A. platae* > *P. canaliculata* > *P. scalaris*). In unstained preparations of isolated corpuscles, the greenish-brown pigmentation of C corpuscles was darker in *P. scalaris* and was slightly more greenish in *A. platae* (as compared with the other species). The corpuscular cover and some inner clumps were alcianophilic in trichrome stained sections of the three species, which is suggestive of Cyanobacteria because they synthesise and excrete glycosaminoglycans (Pereira et al., 2009). A similar conclusion can be inferred from the fact that a generalised cyanobacterial 16S rRNA probe recognises *in situ* the C and K corpuscles of the digestive glands of *P. canaliculata*, *P. scalaris*, and *A. platae* from Lake Regatas. Also, as previously described for *P. canaliculata* (Koch et al., 2006), TEM of C corpuscles revealed numerous electron-dense clumps in an electron-lucent matrix in all the studied species, together with small vesicles and irregularly arranged membrane stacks, but lacking organelles and typical thylakoid structures. Furthermore, an electron-dense wall delimited the C corpuscles in the three species. Intriguingly, this wall occasionally showed small pores in some corpuscles of the three species (only shown for *A. platae* in Fig. 2C). In some cases (Fig. 2A), a membrane appear detached from the external wall, suggesting the existence of a plasma membrane, as it was shown in a previous study of laboratory-raised *P. canaliculata* (Koch et al., 2006). No nuclei were observed in C corpuscles of any of the species studied.

K corpuscles of the three species, instead, had multiple concentric lamellae formed by an electron-dense fibrogranular material and no inner membranes and were often associated with pyramidal cells of the digestive gland, but they differed significantly in length, width, and in the length/width ratio (Table 1). Interestingly, however, lysozyme treatment provoked degradation of isolated C and K corpuscles from the three species, which strongly suggests that peptidoglycan is an important structural element of the wall of C corpuscles and the multiple lamellae of K corpuscles. This finding suggests a bacterial origin of both corpuscle types.

### Mode of transmission of the endosymbionts

Leaving apart K corpuscles, because of the uncertainties on their biological meaning, the finding of C corpuscles in the digestive gland of the major clades of the Ampullariidae suggests their universal occurrence in the family. Such universal occurrence could be explained if the symbiont would have co-evolved with the host, provided (1) the symbiotic association occurred early in the evolution of the Ampullariidae and (2) the endosymbiont was transmitted vertically in the host’s populations.

The predominance of maternal (Koch, Vega & Castro-Vazquez, 2017) versus lateral transmission of the endosymbiont/s in their hosts had not been evaluated before. Indeed, there is evidence of maternal (= vertical) transmission of the endosymbiont (Koch, Vega & Castro-Vazquez, 2017), and therefore the widespread occurrence of the endosymbiotic elements in the major clades of Ampullariidae (Hayes, Cowie & Thiengo, 2009) may be explained by a common ancestral association. However, lateral transmission may continue to some extent in maternally transmitted symbioses (Sachs, Essenberg & Turcotte, 2011). Indeed, the faecal elimination of corpuscles (Castro-Vazquez et al., 2002) and their long persistence in the environment after faecal elimination (Koch et al., 2006) makes lateral transmission possible. Nevertheless, the fact that the C endosymbionts differed amongst the different ampullariid hosts (in size, pigmentation, and degree of occupancy of the tubuloacini), suggests that endosymbionts would have diverged during the co-evolution with their hosts.

## Conclusions

The three species show C and K corpuscles associated with columnar and pyramidal cells, respectively, of the tubuloacinar digestive glands. It is notable that both types of corpuscles together occupy from one-fourth to one-fifth of the tissue area in the three host species. C corpuscles stain positively with Alcian Blue and are delimited by a distinct wall. K corpuscles are dark-brown multilamellar bodies in which no distinct membranes or walls like those of C corpuscles were observed. However, both corpuscle types were sensitive to lysozyme digestion, indicating that peptidoglycans were an integral part of their covers. Also, both C and K corpuscles in the three studied species hybridised with a generalised cyanobacterial/chloroplast probe for 16S rRNA. These data confirm and extend previous studies on *P. canaliculata* in which the endosymbiotic nature of C and K corpuscles were first proposed. We further propose that the endosymbiotic corpuscles are related to the Cyanobacteria/chloroplasts clade.

The known distribution of C and K corpuscles in the major clades of Ampullariidae suggests they may be universally distributed in this family. In a hypothetical scenario, the primordial host may have been an aposymbiotic ancestor to the Ampullariidae, a caenogastropod that lived in the Gondwana supercontinent (Berthold, 1989; Hayes, Cowie & Thiengo, 2009) before its breakup 185–100 Mya (Veevers, 2004). However, several aspects related to this working hypothesis are worth to be explored. For example, the sequences of some marker genes of the endosymbionts such as the 16S rRNA gene, as well as other genomic and proteomic studies would disclose whether the observed differences in the symbiotic corpuscles were only phenotypical and would also disclose the high-rank phylogenetic position of the endosymbiont. Finally, it would be interesting to compare the endosymbiont phylogeny in the major clades of the Ampullariidae (Hayes, Cowie & Thiengo, 2009) to explore the possible co-evolution of the symbiotic consortium.

##  Supplemental Information

10.7717/peerj.8125/supp-1 Figure S1 Flow cytometry and sorting of C and K corpuscles from a mixed suspension of C and K corpuscles obtained from a digestive gland of *Pomacea canaliculata* (A) Dot plot (30,000 events) using forward light scatter (FSC) and side light scatter (SSC). (B) Dot plots of autofluorescence in channels 1 (660 nm) versus 2 (695 nm) of corpuscles in the frame depicted in panel (A). The corpuscles are distributed in two parallel regions, which were framed, sorted, and then microscopically controlled thereafter. (C–F) Sorting of corpuscles in the lower frame showed that 96.7% were C corpuscles (C and E), whereas sorting of those in the upper frame showed that 99.4% were K corpuscles (D and F).

10.7717/peerj.8125/supp-2 Figure S2 Negative control of FISH with a CYA361 rRNA probe, which was not labelled with digoxigenin C and K corpuscles are indicated in merged DIC and fluorescent micrographs of digestive gland sections of *Pomacea canaliculata* (A), *Pomacea scalaris* (B) and *Asolene platae* (C). Abbreviations: c, C corpuscles; k, K corpuscles. Scale bar represents 50 μ m for all panels.

## Additional Information and Declarations

### Field Study Permissions

The following information was supplied relating to field study approvals (i.e., approving body and any reference numbers): Collection of the studied gastropods does not require specific permits. Indeed, both Pomacea canaliculata and *P. scalaris* are invasive species, while Asolene platae, which has not been reported to be invasive, appears in the IUCN Red List as a ’least concern species’ (listed as its junior synonym A. pulchella: www.iucnredlist.org/species/188886/8657832).

### Data Availability

The following information was supplied regarding data availability: Raw data is available at Figshare: Dellagnola, Federico; Rodriguez, Cristian; Castro-Vazquez, Alfredo; Vega, Israel (2019): A Multiple Comparative Study of Putative Endosymbionts in Three Coexisting Apple Snail Species. figshare. Dataset. https://doi.org/10.6084/m9.figshare.8233094.
