# Supplementary figures and images for: A multiple comparative study of putative endosymbionts in three coexisting apple snail species
Source: PeerJ. 2019 Dec 6;7:e8125. doi: 10.7717/peerj.8125 (PMC6901009; doi:10.7717/peerj.8125)

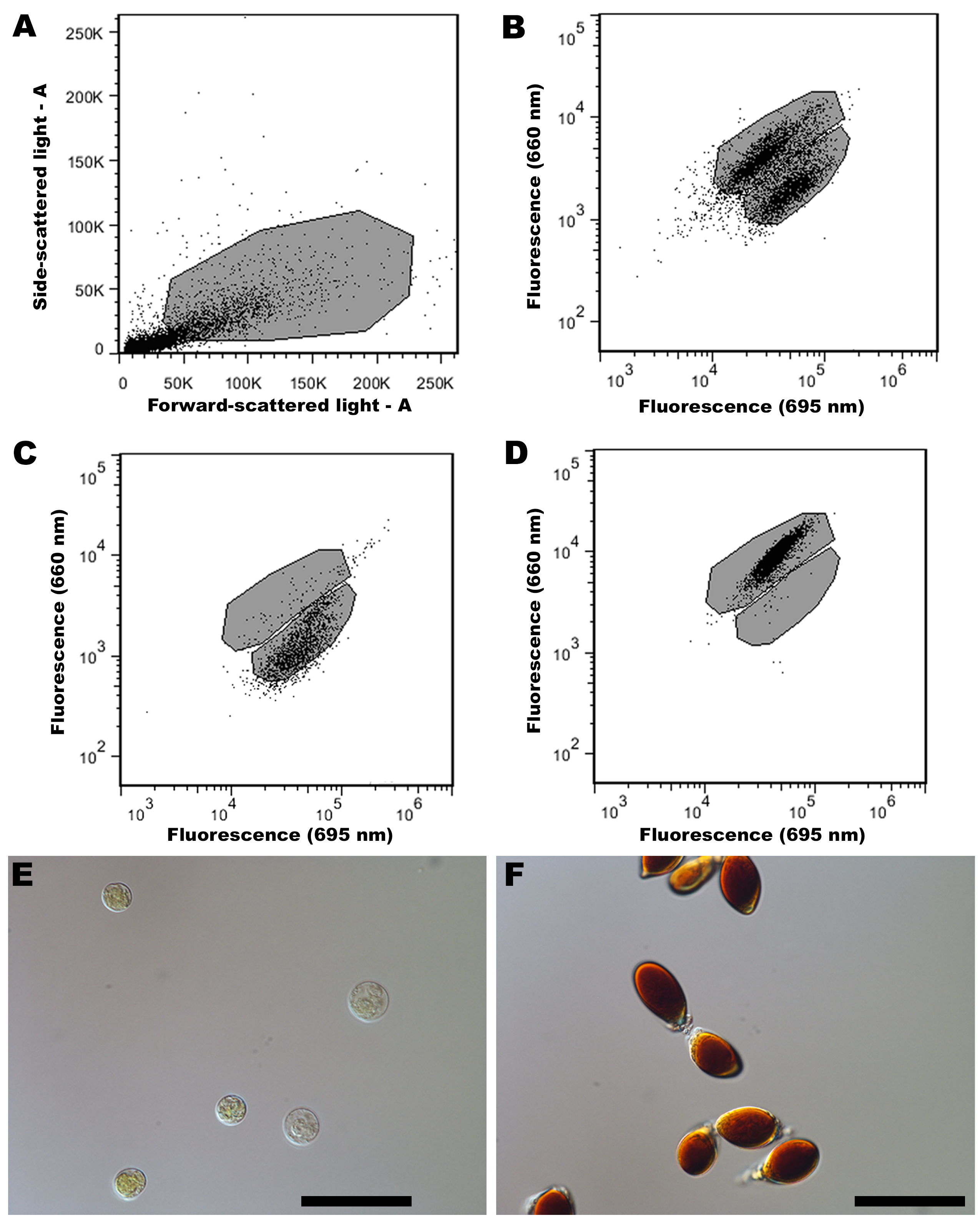

Supplement: Figure S1 — (A) Dot plot (30,000 events) using forward light scatter (FSC) and side light scatter (SSC). (B) Dot plots of autofluorescence in channels 1 (660 nm) versus 2 (695 nm) of corpuscles in the frame depicted in panel (A). The corpuscles are distributed in two parallel regions, which were framed, sorted, and then microscopically controlled thereafter. (C–F) Sorting of corpuscles in the lower frame showed that 96.7% were C corpuscles (C and E), whereas sorting of those in the upper frame showed that 99.4% were K corpuscles (D and F). [file peerj-07-8125-s001.png]

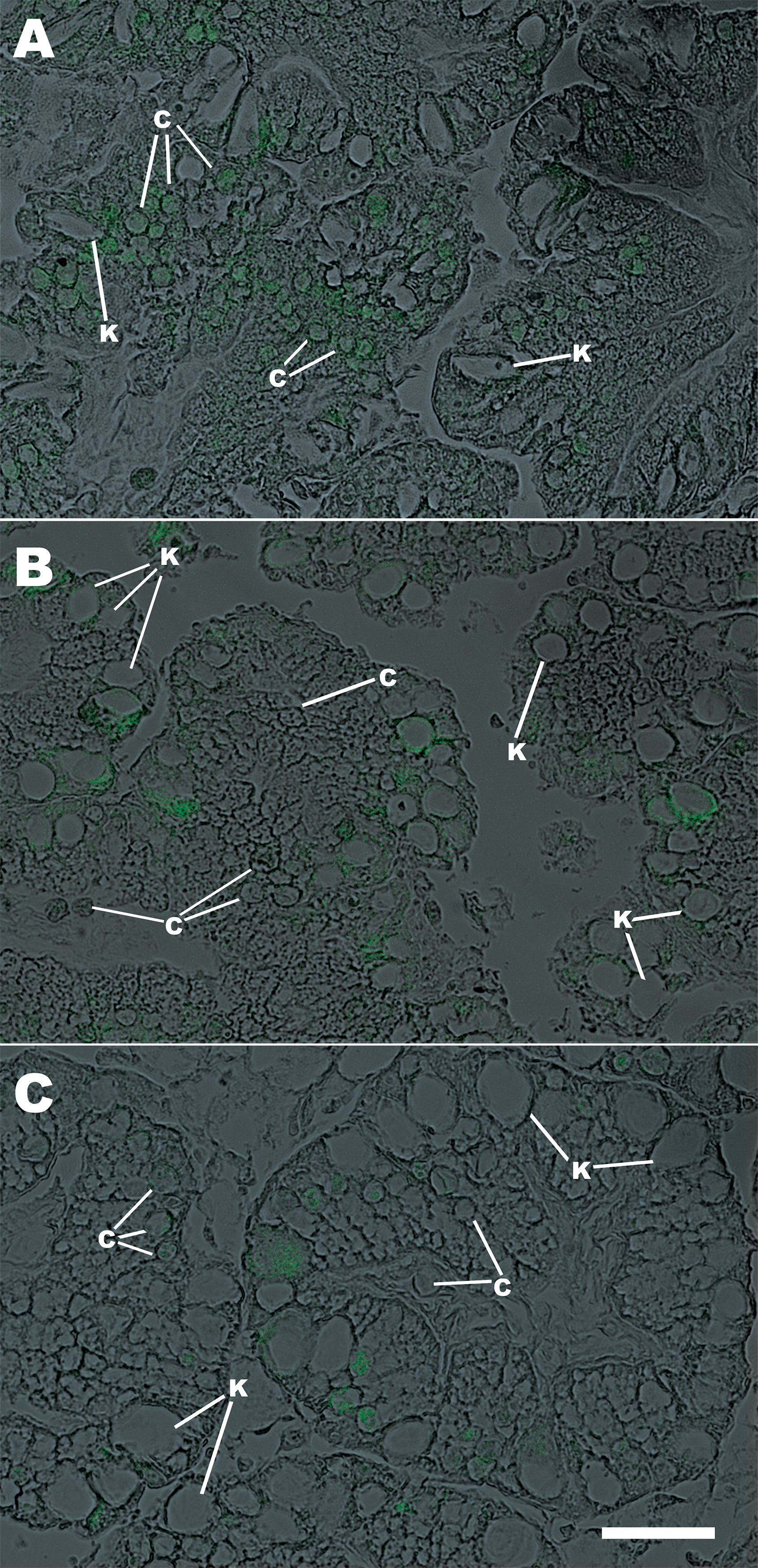

Supplement: Figure S2 — C and K corpuscles are indicated in merged DIC and fluorescent micrographs of digestive gland sections of Pomacea canaliculata (A), Pomacea scalaris (B) and Asolene platae (C). Abbreviations: c, C corpuscles; k, K corpuscles. Scale bar represents 50 μ m for all panels. [file peerj-07-8125-s002.png]
